# Acknowledgement to reviewers 2014/2015

**DOI:** 10.1186/s40634-015-0036-y

**Published:** 2016-01-12

**Authors:** Henning Madry

**Affiliations:** Center of Experimental Orthopaedics, Saarland University Medical Center and Saarland University, Bldg 37, Kirrbergerstr. 100, D-66421 Homburg, Germany

The Journal of Experimental Orthopaedics is very fortunate to have excellent reviewers who kindly agree and spend their time reviewing manuscripts for the journal. Our reviewers work hard to ensure a rapid, rigorous and fair peer review process of each manuscript. Such peer review, focusing solely on the scientific integrity to determine the quality of the research submitted, is very critical to the success of our young journal.

I wish to thank very much those reviewers who provided their time and expertise in evaluating manuscripts for the Journal of Experimental Orthopaedics in 2014/2015.

Sincerely,

Henning Madry

Editor in Chief

**Reviewers 2014/2015**

Ashraf Abdelkafy, Ismailia, Egypt

Roy Altman, Los Angeles, United States of America

Olufemi Ayeni, Hamilton, Canada

Magali Cucchiarini, Homburg, Germany

Nikica Darabos, Zagreb, Croatia

Laura De Girolamo, Milan, Italy

Patricia Díaz Rodríguez, Troy, United States of America

Sabrina Ehnert, Tübingen, Germany

Muhammad Farooq Rai, St Louis, United States of America

Liang Gao, Homburg, Germany

Alan Getgood, Cambridge, United Kingdom

Lars Goebel, Homburg, Germany

Enrique Goméz-Barrena, Madrid, Spain

Tobias Gotterbarm, Heidelberg, Germany

Sibylle Grad, Davos, Switzerland

Farshid Guilak, Durham, United States of America

Alan Hargens, San Diego, United States of America

Emily Hu, Palo Alto, United States of America

Clark Hung, New York, United States of America

Christof Hurschler, Hannover, Germany

Tao Ke, Homburg, Germany

Tomonori Kenmoku, Kanagawa, Japan

Clemens Koesters, Münster, Germany

Elizaveta Kon, Bologna, Italy

Sebastian Kopf, Berlin, Germany

Alexis Lion, Luxembourg, Luxembourg

Christopher Little, St Leonards, Australia

Punyawan Lumpaopong, Phitsanulok, Thailand

Marina Macias-Silva, México D. F., México

Laurent Malisoux, Luxembourg, Luxembourg

Hermann Mayr, München, Germany

Babak Moradi, Heidelberg, Germany

Caroline Mouton, Strassen, Luxembourg

Jason Mussell, New Orleans, United States of America

Koichi Nakagawa, Sakura-shi, Chiba, Japan

Norimasa Nakamura, Osaka, Japan

Sven Nebelung, Aachen, Germany

Geoffroy Nourissat, Paris, France

Patrick Orth, Homburg, Germany

Dietrich Pape, Luxembourg, Luxembourg

Stephen Parada, Fort Gordon, United States of America

Silvia Pianigiani, Milan, Italy

Bernd Rolauffs, Tübingen, Germany

Claudio Rosso, Basel, Switzerland

Ryan Rubin, New Orleans, United States of America

Gian Salzmann, Zurich, Switzerland

Jose Sanhudo, Porto Alegre, Brazil

John Segreti, Chicago, United States of America

Romain Seil, Luxembourg, Luxembourg

Lori Setton, Durham, United States of America

Lazar Sijak, Belgrade, Serbia

Daniel Theisen, Luxembourg, Luxembourg

Jordan Trafimow, Chicago, United States of America

Ronald van Heerwaarden, Woerden, The Netherlands

Francesca Vannini, Bologna, Italy

Peter Verdonk, Antwerp, Belgium

William Walsh, Randwick, Australia

